# Regulatory role of capsaicin-sensitive peptidergic sensory nerves in the proteoglycan-induced autoimmune arthritis model of the mouse

**DOI:** 10.1186/s12974-018-1364-5

**Published:** 2018-12-03

**Authors:** Ádám Horváth, Éva Borbély, Kata Bölcskei, Nikolett Szentes, Tamás Kiss, Mátyás Belák, Tibor Rauch, Tibor Glant, Róza Zákány, Tamás Juhász, Edina Karanyicz, Ferenc Boldizsár, Zsuzsanna Helyes, Bálint Botz

**Affiliations:** 1 Department of Pharmacology and Pharmacotherapy, University of Pécs Medical School, Szigeti u. 12, Pécs, 7624 Hungary; 2 János Szentágothai Research Centre, Molecular Pharmacology Research Team and Centre for Neuroscience, University of Pécs, Pécs, Hungary; 3 Department of Pharmacology and Pharmacotherapy, National Brain Research Program 20017-1.2.1-NKP-2017-00002, Chronic Pain Research Group, University of Pécs Medical School, Pécs, Hungary; 4 Department of Orthopedic Surgery, Section of Molecular Medicine, Rush University Medical Center, Chicago, USA; 5 Department of Anatomy, Histology, and Embryology, University of Debrecen, Debrecen, Hungary; 6 Medical School, Department of Immunology, University of Pécs, Pécs, Hungary; 7 Medical School, Department of Radiology, University of Pécs, Pécs, Hungary

**Keywords:** Rheumatoid arthritis, Mouse model, Experimental arthritis, Neurogenic inflammation, Nociception

## Abstract

**Objective:**

The regulatory role of capsaicin-sensitive peptidergic sensory nerves has been shown in acute inflammation, but little is known about their involvement in T/B-cell driven autoimmune arthritis. This study integratively characterized the function of these nerve endings in the proteoglycan-induced chronic arthritis (PGIA), a translational model of rheumatoid arthritis.

**Methods:**

Peptidergic afferents were defunctionalized by resiniferatoxin (RTX) pretreatment in BALB/c mice, PGIA was induced by repeated antigen challenges. Hind paw volume, arthritis severity, grasping ability and the mechanonociceptive threshold were monitored during the 17-week experiment. Myeloperoxidase activity, vascular leakage and bone turnover were evaluated by in vivo optical imaging. Bone morphology was assessed using micro-CT, the intertarsal small joints were processed for histopathological analysis.

**Results:**

Following desensitization of the capsaicin-sensitive afferents, ankle edema, arthritis severity and mechanical hyperalgesia were markedly diminished. Myeloperoxidase activity was lower in the early, but increased in the late phase, whilst plasma leakage and bone turnover were not altered. Desensitized mice displayed similar bone spurs and erosions, but increased trabecular thickness of the tibia and bony ankylosis of the spine. Intertarsal cartilage thickness was not altered in the model, but desensitization increased this parameter in both the non-arthritic and arthritic groups.

**Conclusion:**

This is the first integrative in vivo functional and morphological characterization of the PGIA mouse model, wherein peptidergic afferents have an important regulatory function. Their overall effect is proinflammatory by increasing acute inflammation, immune cell activity and pain. Meanwhile, their activation decreases spinal ankylosis, arthritis-induced altered trabecularity, and cartilage thickness in small joints.

**Electronic supplementary material:**

The online version of this article (10.1186/s12974-018-1364-5) contains supplementary material, which is available to authorized users.

## Introduction

Rheumatoid arthritis (RA) is the most prevalent autoinflammatory joint disease that results in a considerable burden on both patients and society. There has been a great improvement in the last two decades in its therapy, almost exclusively due to the introduction of targeted monoclonal antibodies [1]. In contrast, in the treatment of chronic, severe pain, limited advances have been made. In chronic pain conditions such as RA long-lasting analgesia and lack of side effects are equally desirable, but most currently available analgesics do not meet these criteria. Hence, it is crucial to explore the complexity of the pathophysiological mechanisms offering novel therapeutic approaches.

It is now established that neuro-immune interactions play a critical role in not only pain and inflammation [2, 3], but also in normal joint and bone homeostasis [4]. Capsaicin-sensitive sensory nerves densely innervate joint capsule and the synovium, and they are crucial for pain perception [5, 6]. These nerves are unique by not only having the classical afferent functions, but they also exert efferent activities [7]. This is orchestrated via the release of multiple mediators, primarily neuropeptides. These nerve endings express transient receptor potential vanilloid 1 (TRPV1) and ankyrin 1 (TRPA1) receptors [8], which are activated and sensitized by numerous exo- and endogenous agents, such as chemicals (capsaicin, or resiniferatoxin; RTX), protons produced in acidic tissue upon inflammation and various inflammatory mediators [9]. The activation results in the release of the aforementioned sensory neuropeptides, including the proinflammatory tachykinins, calcitonin gene-related peptide (CGRP), vasoactive intestinal polypeptide (VIP), and pituitary adenylate cyclase-activating polypeptide (PACAP), that facilitate vasodilation and immune cell recruitment (neurogenic inflammation). Meanwhile, anti-inflammatory and analgesic neuropeptides like somatostatin are also simultaneously released [10]. Numerous studies have proven that the dysregulation of proinflammatory peptide levels occurs in joint tissues and correlates with the severity of RA [11–15]. Moreover, it was also reported that abnormally high levels of the anti-inflammatory somatostatin produced by a somatostatinoma alleviated RA symptoms [16].

The cartilage proteoglycan (PG aggrecan)-induced arthritis (PGIA) in BALB/c mice is a complex model of RA, controlled by major histocompatibility complex (MHC), T cell-dependent, and autoantibody-mediated autoimmune disease [17, 18]. PGIA is a systemic model affecting not only the joints of extremities, but also the axial skeleton. The progression of the disease is also comparable to that of the human condition. The initial phase is characterized by acute inflammatory edema and synovitis, which is followed by bone and cartilage resorption, and it finally progresses into widespread ankylosis of the joints. Furthermore, the multiple genetic loci involved in the development of PGIA are syntenic with the RA risk loci, as proven by genome-wide association studies [19]. Type II collagen and PG are the major components of the articular cartilage, the tissue which is a target of cellular and humoral autoimmunity in RA patients [20].

The concept of peripheral sensory-immune interactions influencing autoinflammatory conditions was introduced decades ago, but our understanding about its exact mechanism still remains fragmentary [21, 22]. We previously demonstrated the crucial role of the capsaicin-sensitive peptidergic afferets in the Complete Freund’s Adjuvant (CFA)-, mast cell tryptase (MCT)-, and K/BxN serum-transfer-induced arthritis models [2, 3, 23]. However, since these models do not properly resemble the complex T and B cell driven mechanism of RA, here we chose the PGIA model, which is a more translational chronic model of RA [24]. Our aims were (1) to characterize the model by functional measurements of pain, joint mobility, and clinical severity, and to follow the progression of the inflammation and bone structure changes by non-invasive imaging methods, as well as (2) to determine the role of the capsaicin-sensitive afferents in these pathophysiological alterations.

## Methods

### Ethical approval

The experiments were performed according to European legislation (Directive 2010/63/EU) and Hungarian Government regulation (40/2013., II. 14.) of the protection of animals used for scientific purposes. The studies were approved by the Ethics Committee on Animal Research of the University of Pécs according to the Ethical Codex of Animal Experiments (license no: BA 02/2000–2/2012) and complied with the recommendations of the International Association for the Study of Pain. The human sample collection was approved by the Institutional Review Board of Rush University Medical Center, Chicago, USA.

### Animals and immunization

Experiments were performed on 8–12-week-old female BALB/c mice. Animals were bred and maintained in the animal facility of the Department of Immunology of the University of Pécs, housed at 24–25 °C ambient temperature, and provided with standard rodent chow and water ad libitum under 12 h light/dark cycles.

PG was isolated, purified, and prepared for immunization from human knee cartilages harvested during joint replacement surgery. In brief, PG was extracted by 4 M guanidinium chloride, centrifuged, partially deglycosylated, and purified as described [25]. BALB/c mice were immunized with 100 μg of core protein of PG in 100 μl of phosphate-buffered saline (PBS; pH 7.4) [20], emulsified with 2 mg of dimethyl-dioctadecyl ammonium bromide (Sigma-Aldrich, St. Louis, MO, USA) in 100 μl of PBS three times intraperitoneally (i.p.) at 3-week intervals [26].

### Resiniferatoxin pretreatment (desensitization)

Repeated pretreatment with RTX (Sigma-Aldrich; 30, 70, 100 μg/kg subcutaneously on 3 consecutive days), an ultrapotent TRPV1 agonist induced a long-lasting defunctionalization of the capsaicin-sensitive peptidergic afferents [2, 3, 27]. A 1 mg/ml stock solution was prepared, which was further diluted with saline to 3, 7, and 10 μg/ml. The volume of administration was 0.1 ml/10 g body weight. 2 weeks after the last RTX administration, the success of the desensitization was verified by the lack of eye-wiping response to capsaicin drops (50 μl, 0.1%) [3].

### Experimental design

Our measurements were carried out after the first i.p. PG injection administered 2 weeks after RTX-pretreatment. The mechanononiceptive threshold and the volume of the hind paws were evaluated once a week from the 1st to the 12th week, neutrophil myeloperoxidase (MPO) activity, and inflammatory vascular leakage were measured from the 6th to the 14th week. Arthritis severity and joint function were assessed three times a week from 1st to the 16th week of the study. Bone remodeling was assessed on week 13 of the immunization, while micro-computed tomography (micro-CT) was performed at the end of the 17-week experiment. Following the micro-CT scans, the animals were euthanized, and their ankle joints were removed for histological processing (Additional file 1: Figure S1).

### Assessment of disease severity and hind limb edema

The volume of the hind paws was measured weekly by plethysmometry (Ugo Basile, Comerio, Italy) [2, 3]. Arthritis severity and hyperemia were also evaluated semiquantitatively three times a week by visually scoring on a scale of 0–4 for each limb (0, no edema or redness; 1, mild edema of the distal paw or at least two digits; 2, moderate swelling of the paw joints distal to the ankle or wrist; 3, marked edema and hyperemia of the paw including the ankle or wrist; 4, severe swelling and redness of the entire paw with joint stiffness) [20, 26, 28]. All the four extremities were scored by inspection and passive moving of the joints; therefore, each mouse was scored from 0 to 16.

### Measurement of mechanonociception and joint function

The mechanonociceptive threshold of the hind paws was measured weekly by dynamic plantar esthesiometry (Ugo Basile). The maximum force was set to 10 g, and the cut-off time was 4 s. Mechanical hyperalgesia was expressed as the percent change compared to the pretreatment controls [2]. Joint function and grasping ability was assessed three times a week by placing the mice on a horizontal wire mesh grid, which was consequently turned over to determine the latency to fall [2]. The cut-off time was 60 s. The percentage of mice which were able to grasp the grid for the whole period of time was plotted as a survival curve [29].

### In vivo bioluminescent imaging of neutrophil MPO activity

Luminol is a chemiluminescent sensor of reactive oxygen species (ROS) that mainly correlates with neutrophil MPO-activity in vivo [30]. A 30 mg/ml PBS solution of luminol (GoldBio, St. Louis, MO, USA) was injected i.p. at a dose of 150 mg/kg. Animals were anesthetized using ketamine (120 mg/kg; Calypsol, Gedeon Richter, Budapest, Hungary) and xylazine (6 mg/kg; Sedaxylan, Eurovet Animal Health, Bladel, The Netherlands). Imaging was performed 10 min post injection using the IVIS Lumina II optical imager (PerkinElmer, Waltham, USA) weekly between the 6th and the 14th weeks of the experiment, as neutrophil MPO-activity is predominantly increased during the acute phase of inflammation. The acquisition time was 120 s, F/stop = 1, and binning = 8. Regions of interest (ROIs) were drawn corresponding to the hind limbs, in which luminescence was expressed as total radiance (total photon flux/s) [31].

### In vivo fluorescent imaging of inflammatory vascular leakage and bone turnover

Inflammatory vascular leakage was evaluated by intravenous (i.v.) injection of IR-676 (Spectrum-Info, Kyiv, Ukraine), a vascular fluorescent dye, which was dissolved in a 5 *w*/*v*% aqueous solution of Kolliphor HS 15 (polyethylene-glycol-15-hydroxystearate; Sigma-Aldrich) and injected into anesthetized mice at a dose of 0.5 mg/kg. Imaging was performed 20 min post injection using the IVIS Lumina II in vivo imaging system (PerkinElmer). Acquisition time was automatic, F/stop = 1, binning = 2, excitation/emission filters were 640/700 nm. The ROIs were applied to the ankle joints, in which fluorescence was expressed as total radiant efficiency ([photons/s/cm^2^/steradian/[μW/cm^2^]) [32].

Arthritic bone remodeling was assessed by OsteoSense 680 EX (PerkinElmer), a fluorescently labeled bisphosphonate in vivo imaging agent, which was injected i.v. (2 nmol/subject) [33]. Measurements were performed using the FMT 2000 fluorescence molecular tomography system 24 h later (PerkinElmer). Three-dimensional (3D) reconstructions of the region of the ankles were made, and ROIs were drawn, in which the amount of the fluorophore was determined in picomoles. Since imaging the spine with the FMT system is technically challenging, bone remodeling in the vertebral column was measured by the IVIS system. The imaging parameters were the following: auto acquisition time, F/Stop = 1, and binning = 2. The excitation and emission filters were 640/700 nm. The ROIs were applied as described earlier, and radiant efficiency was expressed and used for further analysis.

### In vivo micro-CT analysis of the ankles and lumbar spine

The right ankles and the lumbar spinal region were repeatedly scanned using a 17.5 μm voxel size by a SkyScan 1176 in vivo micro-CT (Bruker, Kontich, Belgium). Changes of bone structure were assessed using the CT Analyzer® software. ROIs of standard size were drawn around the respective regions, in which bone volume (μm^3^), bone surface (μm^2^), average thickness (mm) and number (1/mm) of bone trabeculae, and the total volume of bone pores (μm^3^) were calculated [2]. The width of each lumbar intervertebral space was manually measured at the ventral aspect of the spinal column (μm), the means of which was used for further analysis.

### Histological processing and assessment of cartilage thickness

The hind limbs of the mice of all the experimental groups were fixed and then decalcified in a solution of 4% ethylenediaminetetraacetic acid (EDTA) for two weeks. Samples were immersed into a pre-made rapid bone decalcification solution (Decalc, HistoLab, Stockholm, Sweden). Decalcified tissue samples were embedded into paraffin, sectioned, and stained with a 0.1% aqueous solution of dimethyl-methylene blue (DMMB) (Sigma-Aldrich). Histological analysis was performed in a blinded fashion. Representative photomicrographs were taken using an Olympus DP72 camera on a Nikon Eclipse E800 microscope (Nikon Corporation, Tokyo, Japan). Images were acquired using CellSense Entry 1.5 software (Olympus, Shinjuku, Tokyo, Japan).

Measurement of cartilage thickness was performed by using Adobe Photoshop version 10.0 software. Since the tibiotarsal joint underwent complete bony ankylosis in both groups, the smaller intertarsal joint was selected for evaluation of the articular cartilage. The average of cartilage thicknesses in different experimental groups were calculated and compared.

### Statistics

Results are expressed as mean ± standard error of mean (SEM). Statistical evaluation was performed by GraphPad Prism®. Mechanical hyperalgesia, paw edema, arthritis scores, the optical imaging of MPO activity, and vascular leakage, as well as the micro-CT imaging of the ankle were evaluated by repeated measures two-way analysis of variance (ANOVA) and Bonferroni’s multiple comparison test. In the case of mechanical hyperalgesia and paw edema, percentage change values were compared between treatment groups. In the case of the grid test, percentage values were assessed by the log rank test. Optical imaging of bone remodeling, the micro-CT imaging of the lumbar spine, and the histological analysis of cartilage thickness were assessed by one-way ANOVA and Tukey’s multiple comparison test. In all cases, *p* < 0.05 was considered significant.

## Results

### Lack of capsaicin-sensitive nerves diminishes arthritic mechanical hyperalgesia, but not grasping ability

**Fig. 1:**
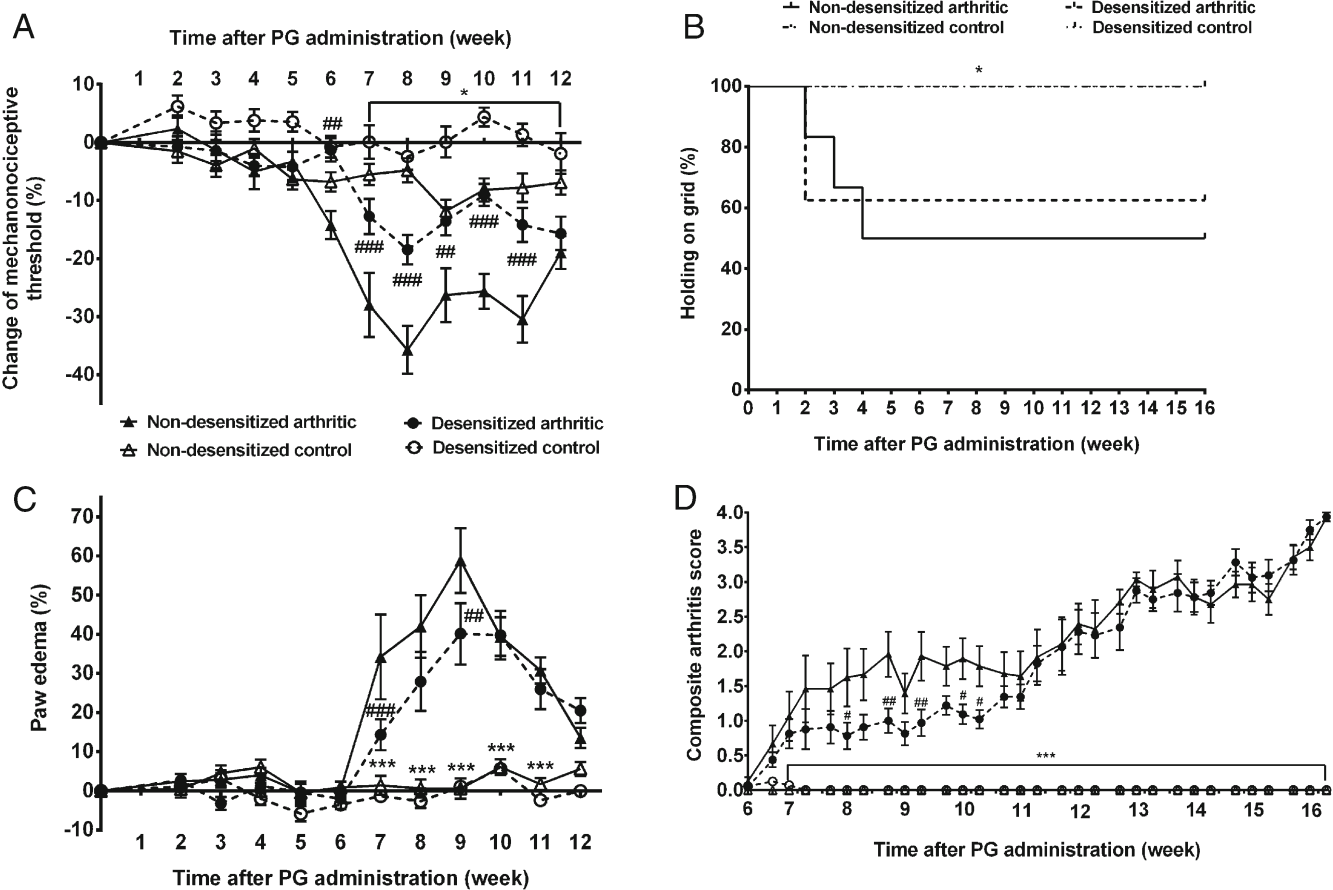
Diminished mechanical hyperalgesia, edema, and arthritis severity score, but retained grasping ability after sensory desensitization. The changes of the hind paw mechanonociceptive threshold (**a**), grasping ability (**b**), hind limb edema (**c**), and the semiquantitative arthritis severity score (**d**). Values are the means ± SEM, *n* = 7–8/group. *p < 0.05, ***p < 0.001 vs. respective non-arthritic controls, ^#^*p* < 0.05, ^##^*p* < 0.01, ^###^*p* < 0.001 vs. non-desensitized arthritic mice. Grasping ability was assessed by log rank test, all other functional results by repeated measures two-way ANOVA

The baseline mechanonociceptive threshold values of the groups did not differ from each other (Additional file 2: Table S1). Mechanical hyperalgesia peaked between the 8th and the 11th week of the experiment at 35% in non-desensitized and 20% in desensitized arthritic mice, remaining significantly lower in the latter group between the 6th and 11th weeks of the experiment (Fig. 1a). Analyzing the percentage of animals which could stay on the horizontal grid for 60 s, the grasping ability of non-desensitized arthritic mice decreased until the 4th week, while in the desensitized arthritic group, its decrease was observed in the 2nd week. Despite the worsening joint ankylosis, 50–60% of the arthritic mice could stay on the grid for 30 s by week 4. However, a significant difference was not seen between these groups (Fig. 1b). The latency values of the grid test are shown in Additional file 3: Table S2, and the results of the statistical analysis in Additional file 4: Table S3.

### Lack of capsaicin-sensitive nerves decreases arthritic edema and inflammation severity score

Hind limb edema reached its peak during the 9th week. The peak paw volume increase was 60% in non-desensitized mice and significantly lower (40%) in desensitized animals between the 7th and 9th weeks (Fig. 1c). The arthritis severity score, also considering limb stiffness and ankylosis increased gradually, and was significantly lower in desensitized mice during the 8th to 10th weeks (Fig. 1d). The statistical analysis is shown in Additional file 4: Table S3.

### Lack of capsaicin-sensitive nerves bidirectionally alter inflammatory MPO activity, but not vascular leakage and bone turnover

**Fig. 2:**
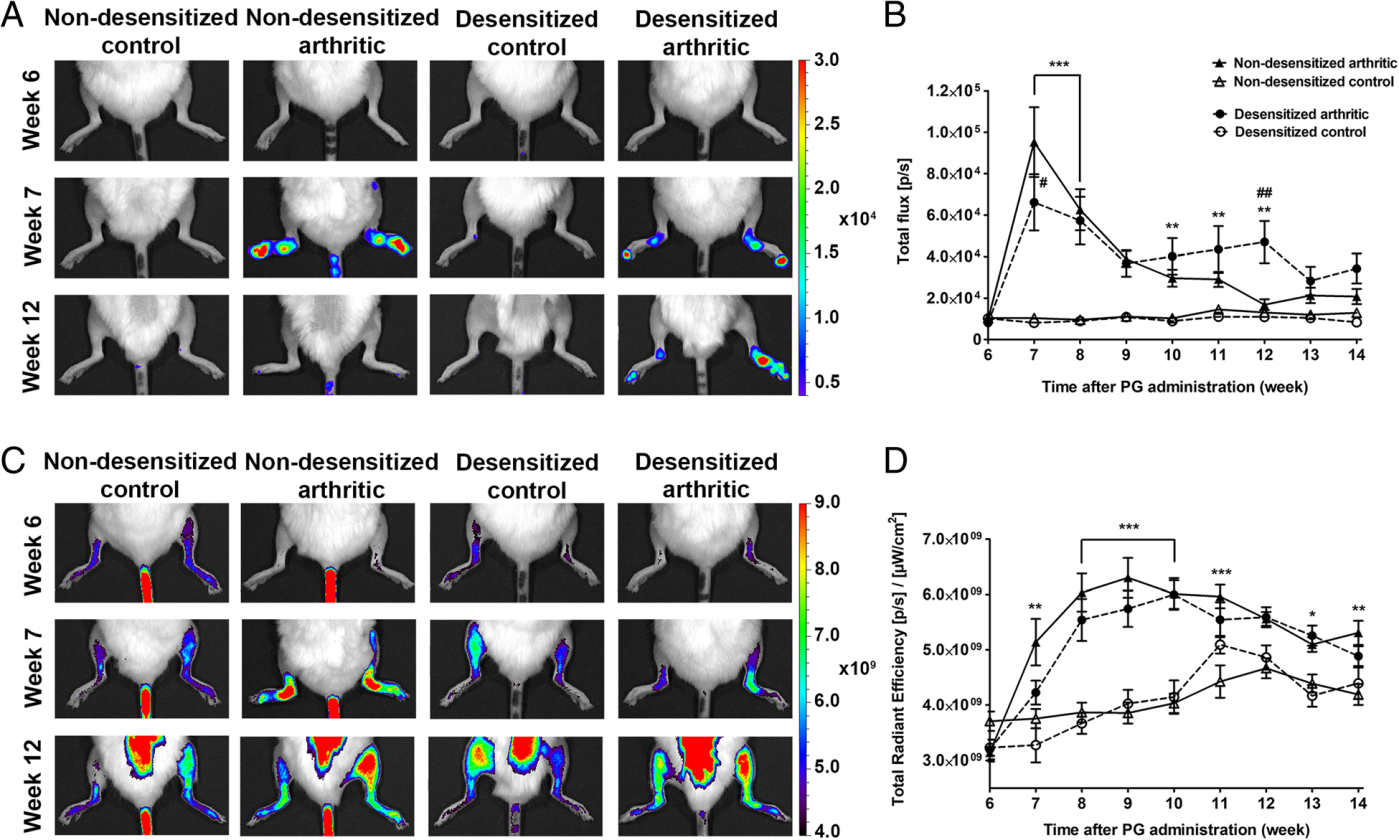
Lower MPO activity in the acute, but greater in the chronic phase of arthritis, without vascular leakage alterations after sensory desensitization. Representative 2D bioluminescence images of MPO activity (**a**), and the quantification of the luminescence in the hind limbs (**b**). Representative 2D fluorescence images of vascular leakage (**c**), and the quantitative evaluation of fluorescence (**d**). Values are the means ± SEM, *n* = 7–8/group. *p < 0.05, **p < 0.01, ***p < 0.001 vs. respective non-arthritic controls, ^#^*p* < 0.05, ^##^*p* < 0.01 vs. non-desensitized arthritic mice. Repeated measures two-way ANOVA was used

**Fig. 3:**
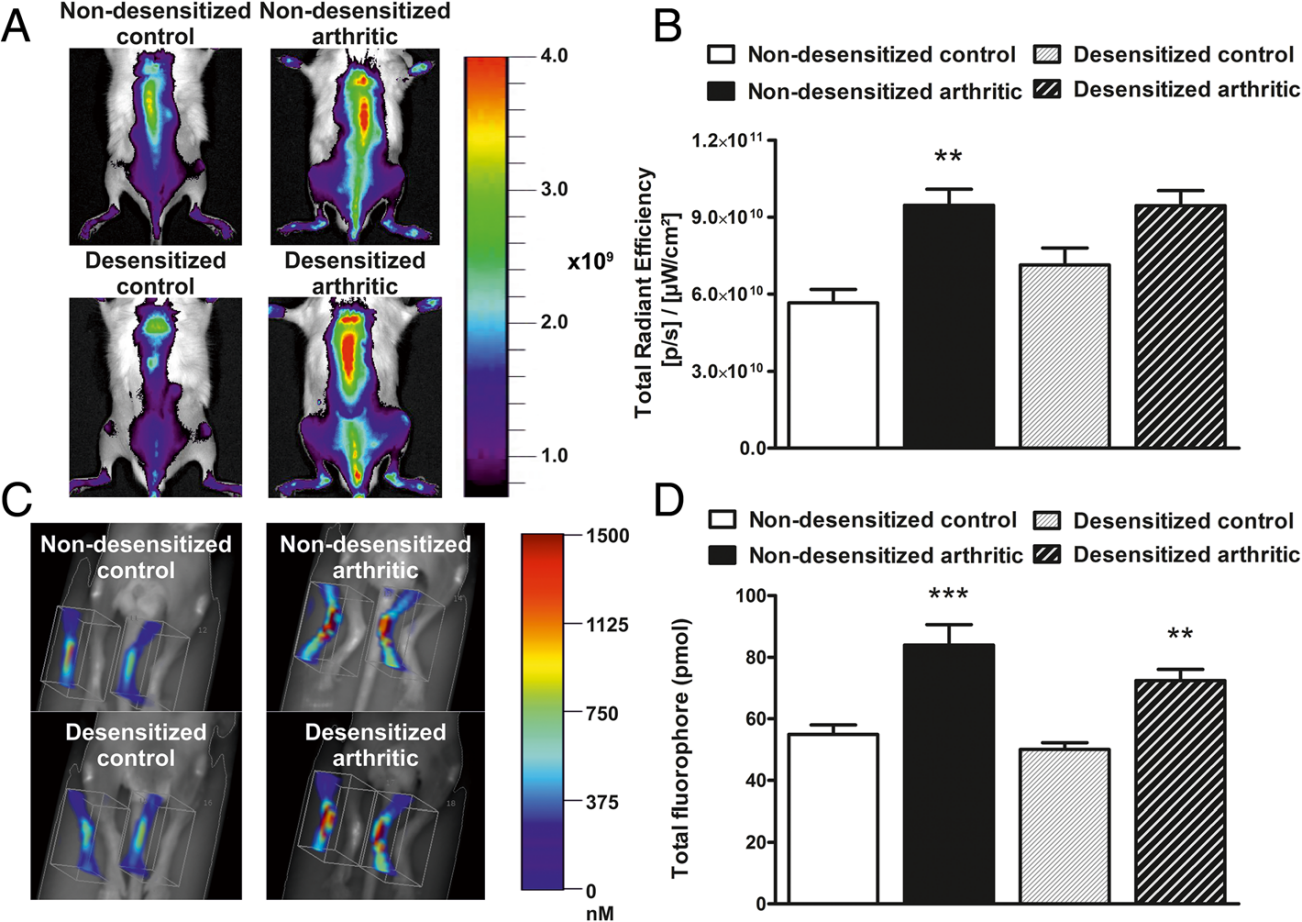
Arthritic bone turnover increase is unaltered after sensory desensitization. Representative 2D epifluorescence images of the spine (**a**), and the quantification of OsteoSense fluorescence (**b**). Representative 3D FMT reconstructions of the ankle joints (**c**) and the quantification of the amount of fluorophore in picomols (**d**). Values are the means ± SEM, *n* = 5–6/group. ^**^p < 0.01, ^***^p < 0.001 vs. respective non-arthritic control. One-way ANOVA was used

ROS production peaked in the 7th week in non-desensitized arthritic mice, and gradually decreased thereafter. In desensitized animals, MPO-activity was significantly lower in the early phase, but remained relatively steady thereafter, and was significantly higher in the 12th week of the study (Fig. 2a–b). In non-desensitized mice, vascular leakage reached its peak during the 9th week. In desensitized mice, the inflammatory vascular permeability increase was similar to that of their controls (Fig. 2c–d). The statistical analysis is shown in Additional file 4: Table S3. Bone remodeling in the 13th week increased significantly in both the spine (Fig. 3a–b) and ankle joints (Fig. 3c–d) of non-desensitized arthritic mice. In desensitized animals, the bone turnover increase was only significant in the ankle.

### Lack of capsaicin-sensitive nerves increases trabecular thickness of the bones of the arthritic ankle joint

**Fig. 4:**
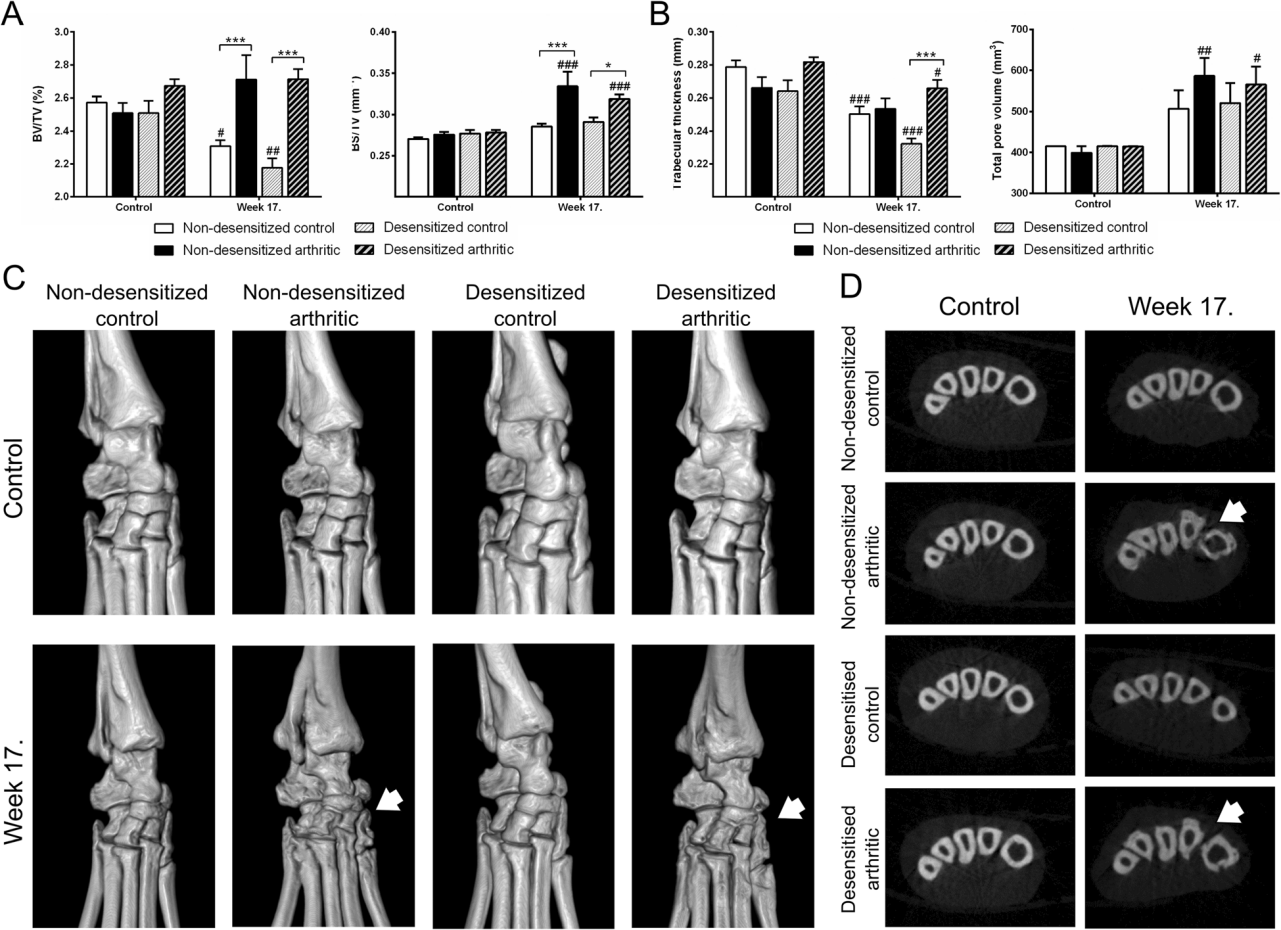
Increased trabecular thickness in the arthritic ankle joint, but unaltered bone spur formation and porosity after sensory desensitization. Changes in bone volume and surface density (BV/TV, BS/TV) (**a**), trabecular thickness, and total pore volume (**b**). Representative 3D CT reconstructions of ankle joints (**c**) and axial CT slices (**d**) demonstrating cortical bone loss and osteophyte formation (cortical irregularities highlighted by arrowheads). Values are the means ± SEM, *n* = 7–8/group. *p < 0.05, ***p < 0.001 vs. respective non-arthritic control, ^#^*p* < 0.05, ^##^*p* < 0.01, ^###^*p* < 0.001 vs. respective initial control. Two-way ANOVA was used

By the 17th week of the experiment, a significant bone volume loss of approximately 10% could be observed in both non-desensitized and desensitized control mice (Fig. 4a), due to the decreased density of the cortical bone, also noticeable on the axial CT slices (Fig. 4d). The bone density in arthritic mice remained unchanged, and thus became significantly higher than in the respective non-arthritic groups by the end of the study, reflecting periarticular osteophyte formation (Fig. 4c–d). The relative bone surface increased significantly in both non-desensitized and desensitized arthritic mice (Fig. 4a). This, coupled with the slightly but significantly increased total pore volume (Fig. 4b), was predominantly caused by inflammatory bone erosions, which could be also observed on the 3D images (Fig. 4c). The mean thickness of the bone trabeculae decreased by 10–12% in the non-arthritic groups, while it remained relatively unchanged in arthritic animals (Fig. 4b). The trabecular thickness was significantly higher only in desensitized arthritic group compared to the desensitized control group.

**Fig. 5:**
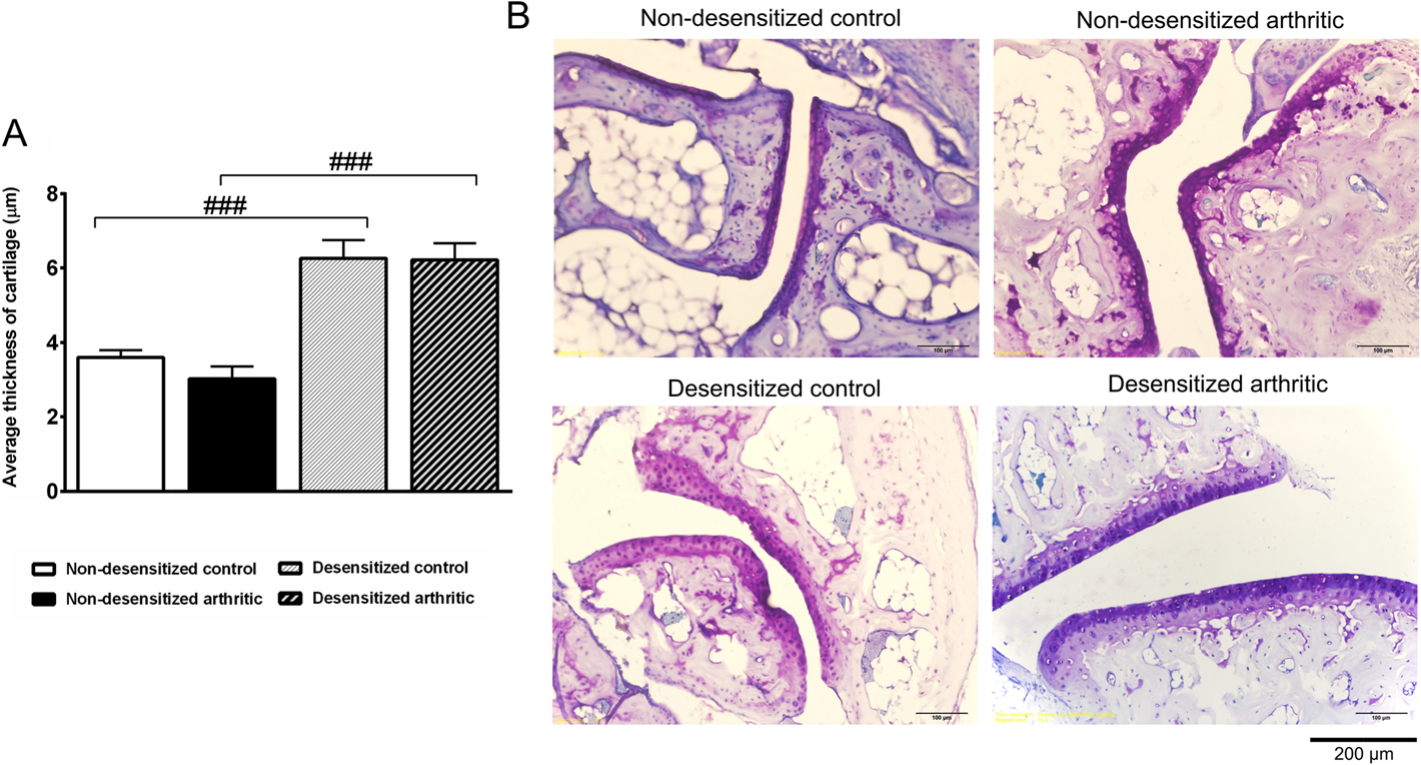
Increased intertarsal cartilage thickness after sensory desensitization. Average thickness of cartilage (μm) (**a**). Representative photomicrographs of the intertarsal joints (**b**). Values are the means ± SEM, *n* = 7–8/group, ^###^*p* < 0.001 vs. respective non-desensitized group. One-way ANOVA was used

Since the tibiotarsal joint underwent complete ankylosis, cartilage could only be objectively assessed in the smaller intertarsal joints at week 17, which were seemingly not affected by the arthritis. However, in the desensitized animals, regardless of the induction of PGIA, a significantly elevated thickness of the intertarsal cartilage was found (Fig. 5a–b).

### Lack of capsaicin-sensitive nerves increases bony ankylosis in the arthritic spine

**Fig. 6:**
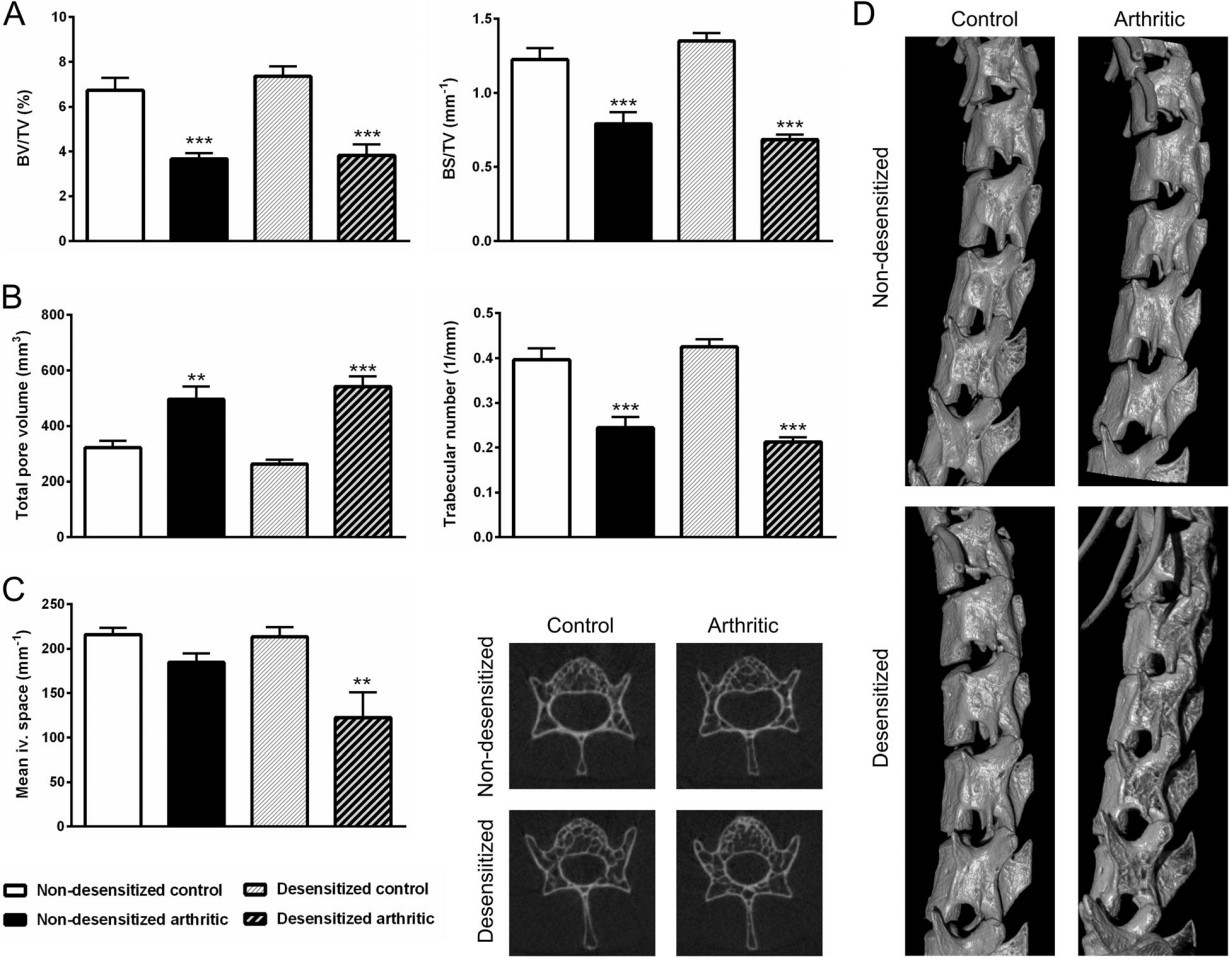
Increased bony ankylosis and unaltered spinal bone remodeling after sensory desensitization. Quantification of bone volume and surface densities (BV/TV, BS/TV) (**a**), total pore volume and the number of trabeculae (**b**). Quantification of the mean intervertebral space, and representative axial CT slices (**c**). Representative 3D reconstructions of the lumbar spine (**d**). Values are the means ± SEM, *n* = 7–8/group. **p < 0.01, ***p < 0.001 vs. respective non-arthritic control. One-way ANOVA was used

Non-desensitized and desensitized arthritic mice displayed a similar decrease in spinal bone density and relative surface by the 17th week of the experiment compared to their respective controls (Fig. 6a). An increased total porosity and a decreased density of bone trabeculae could also be observed (Fig. 6b). The mean width of the lumbar intervertebral spaces was significantly lower by approximately 40% in desensitized arthritic mice compared to their non-desensitized controls (Fig. 6c). This was due to the also visually apparent bony ankylosis (Fig. 6d).

## Discussion

The current work presented two lines of novel data on the PGIA mouse model of RA. The first was the complex characterization of the different stages of the chronic PGIA model of the mouse using integrative methodologies. The PGIA model has been known as a T cell-dependent chronic model of RA, mimicking several molecular pathways of the human disease including autoantibody-production and the cytokine profile [20]. It is also known that ankylosis of the affected joints occurs during the late phase, and both the joints and the spine demonstrate evidence of simultaneous bone erosion and neoformation [17]. Although arthritic pain is a critical aspect of RA, nociceptive responses have not been studied in this model. Ours was the first study to show that PGIA induces a long-lasting mechanical hyperalgesia, and thus it can be suggested as an appropriate model of chronic arthritic pain. A limitation of our study is that the nocifensive behavior could not be investigated during the total experimental period due to the ankylosis of the ankle joint after the 3rd month. We also provided compelling evidence of the complex effects of PGIA on bone metabolism and architecture using novel non-invasive imaging methods. Arthritic mice demonstrate increased bone turnover in the hind paws, coupled with the development of periarticular osteophytes and erosions. The significant decrease in bone density in the control mice demonstrates the critical importance of using age-matched controls in studies investigating bone structural changes in RA models.

The other important novelty of our results is that we provide the first evidence about the pivotal role of the innervating capsaicin-sensitive sensory afferents in the T and B lymphocyte-dependent disease-mimicking model of RA. We showed that selective defunctionalization of these nerves leads to decreased arthritis severity, edema, and hyperalgesia. However, its effect on neutrophil ROS production follows an interesting biphasic pattern, demonstrated by MPO activity that is lower in the early, but higher in the late phase of arthritis. The functional implication of the late neutrophil activation is not clear as we did not see a corresponding worsening of functional parameters. On the other hand, desensitization had no protective effect on the arthritis-induced changes in bone turnover or bone morphometry.

The model induces a complete bony ankylosis of the ankle joints, while the intertarsal small joints remain undamaged. An unexpected result of our study was that RTX defunctionalization increased cartilage thickness in these joints. It has been demonstrated that peptidergic sensory nerves penetrate the outer layers of articular cartilage and modulate chondrocyte differentiation, mainly via the release of calcitonin gene-related peptide (CGRP) [4]. Thus, it can be speculated that the selective deactivation of these fibers interferes with the homeostasis of the articular cartilage.

Our present study demonstrated noteworthy differences when compared to our earlier experiments using the CFA-induced, K/BxN serum-transfer, and MCT models of arthritis. In both the CFA-induced and serum-transfer models, RTX-pretreatment aggravated arthritis severity but reduced inflammatory hyperalgesia [2, 3]. However, in the MCT model, joint hyperemia, and spontaneous pain but not edema were diminished in desensitized animals [23]. These essentially contradictory results of the earlier and the current experiments are at least partly due to distinct mechanisms and therefore, the limited translational value of the previously used models. For example, although the CFA-induced rat arthritis model was originally described as a RA disease model, it has been proven to rather model reactive arthritides with many features not found in human RA [24, 34]. Arthritis in K/BxN mice is triggered by autoreactive T cells and maintained by the autoantibody production of B-cells, but serum-transfer arthritis is a transient T/B-cell-independent model mimicking primarily the innate immune response to joint-specific autoantibodies [35, 36]. In a similar way, the MCT model evoked solely by a mast-cell-specific protease represents only one facet of the complexity of autoimmune arthritis [37]. The PGIA model is the most translational RA model in term of adequately resembling all aspects of the autoinflammatory response. In summary, the observed differences highlight the importance of desensitization in a T/B cell-driven arthritis model. Of note, we cannot exclude that lymphocytes were directly affected by the RTX treatment. The extraneural expression of TRP receptors has recently became a topic of interest, and it has been proven that functional TRPV1 is expressed on T (but not B) cells, contributing to their pro-inflammatory response [38, 39]. On the other hand, we have to mention that while the effect of desensitization on TRPV1 present on T cells has not been addressed, our own earlier studies involving other extraneural tissues, such as skin or oral mucosa, failed to demonstrate an effect of RTX-pretreatment on receptors present in such tissues [40]. We have previously shown that TRPV1 and TRPA1 gene-deficient mice show diminished inflammation, nociception, and histological damage in CFA-induced model [41, 42]. Moreover, in the K/BxN-serum transfer arthritis model reduced hyperalgesia but increased edema was detected in TRPV1 gene-deleted mice, while no differences were revealed in TRPA1 gene-deficient animals [43]. These findings, taken together with the current results indicate that TRP channel activation aggravates local inflammation via the release of pro-inflammatory mediators, while it is also critical for inflammatory pain. Concerning a direct effect on the pain component, it has to be pointed out that the involvement of capsaicin-sensitive primary afferents in mechanonociception, mechanical hyperalgesia, and allodynia in models of inflammatory, neuropathic, or cancer pain had been extensively investigated. Several studies could not confirm a role for this subpopulation of sensory neurons in mechanical hyperalgesia in contrast with thermal hyperalgesia and spontaneous pain in these conditions [44–49], which might be due to major differences in peripheral and central sensitization mechanisms in these chronic pathophysiological processes. However, in our long-lasting arthritis model with mixed inflammatory, neuropathic, and degenerative mechanisms, we found a markedly diminished mechanical hyperalgesia in desensitized arthritic animals, which points to the feasibility of targeting TRPV1 channels and TRPV1-expression sensory nerves for analgesia at the periphery.

Previous studies have demonstrated the importance of certain pro- and anti-inflammatory sensory neuropeptides in arthritis models [10, 31, 50], while the overall effect of sensory desensitization was found to be primarily anti-inflammatory and analgesic in our current study. The delayed decrease of MPO-activity has previously also been observed in the K/BxN serum transfer model, where mice deficient in PACAP, a major vasoactive peptide mediator, showed a similarly increased neutrophil ROS production in the late phase of arthritis [31].

The involvement of the axial skeleton is a unique feature of PGIA, characterized by a robust spondyloarthropathy, syndesmophyte formation, and intervertebral disc degeneration; hence it is also utilized as a model for ankylosing spondylitis [51–53]. Our study demonstrated narrowing of the intervertebral space, an indirect sign of disc degeneration, is increased in desensitized arthritic mice, while other aspects of bone structural changes are similar to those of non-desensitized animals. This suggests that peptidergic afferents have an overall protective effect on PGIA-induced spondyloarthropathy.

## Conclusions

It can be concluded that capsaicin-sensitive sensory nerves play an important regulatory role in arthritic pain and inflammation in the PGIA model of RA. The impact of these afferents goes well beyond acute inflammation and nociception; they also have a complex influence on the skeletal structural changes. Further studies are needed to identify the specific key mediators released from these nerves, their targets and the signaling pathways involved in the pathophysiological mechanisms of the disease. Special emphasis should be put on pain-related processes, since chronic arthritic pain is still an unmet medical need. The pro-inflammatory and algogenic functions of these afferents suggest that blocking these nerve terminals at the periphery might be a promising treatment option.

## Additional files

Additional file 1: **Figure S1.** Experimental design. (TIF 2950 kb)

Additional file 2: **Table S1.** Mean and SEM values of mechanonociceptive thresholds measured with the DPA of each experimental group. (DOCX 15 kb)

Additional file 3: **Table S2.** Mean and SEM values of the latency to fall from the horizontal grid of each experimental group. (DOCX 21 kb)

Additional file 4: **Table S3.** Results of the statistical analysis with repeated measures two-way ANOVA of mechanical hyperalgesia, paw edema, arthritis score, and optical imaging data of MPO activity and vascular leakage. (DOCX 14 kb)

## Abbreviations

ANOVA: Analysis of variance
CFA: Complete Freund’s adjuvant
CGRP: Calcitonin gene-related peptide
DMMB: Dimethyl-methylene blue
EDTA: Ethylenediaminetetraacetic acid
MCT: Mast cell tryptase
MHC: Major histocompatibility complex
micro-CT: Micro-computed tomography
MPO: Myeloperoxidase
PACAP: Pituitary adenylate cyclase-activating polypeptide
PBS: Phosphate-buffered saline
PG: Proteoglycan
PGIA: Proteoglycan-induced arthritis
RA: Rheumatoid arthritis
ROI: Regions of interest
ROS: Reactive oxygen species
RTX: Resiniferatoxin
SEM: Standard error of mean
TRPA1: Transient receptor potential ankyrin 1
TRPV1: Transient receptor potential vanilloid 1
VIP: Vasoactive intestinal polypeptide
