# Supplementary material for: Regulatory role of capsaicin-sensitive peptidergic sensory nerves in the proteoglycan-induced autoimmune arthritis model of the mouse
Source: J Neuroinflammation. 2018 Dec 3;15:335. doi: 10.1186/s12974-018-1364-5 (PMC6276168; doi:10.1186/s12974-018-1364-5)
Supplement: Supplementary file 2 — Table S1. Mean and SEM values of mechanonociceptive thresholds measured with the DPA of each experimental group. (DOCX 15 kb) [file 12974_2018_1364_MOESM2_ESM.docx]

**Table S1: Mean and SEM values of mechanonociceptive thresholds measured with the DPA of each experimental group.**

| **weeks** | **non-desensitized control** | | **non-desensitized PGIA** | | **desensitized control** | | **desensitized PGIA** | |
| --- | --- | --- | --- | --- | --- | --- | --- | --- |
|  | **mean** | **SEM** | **mean** | **SEM** | **mean** | **SEM** | **mean** | **SEM** |
| **0** | 9.4175 | 0.1095188 | 8.978572 | 0.0921141 | 8.709723 | 0.1109823 | 9.139063 | 0.1019714 |
| **2** | 9.259375 | 0.1508268 | 9.172143 | 0.1791775 | 9.229445 | 0.1439873 | 9.0625 | 0.158389 |
| **3** | 8.970625 | 0.1152225 | 8.835714 | 0.2365218 | 8.982223 | 0.15092 | 8.996875 | 0.1403529 |
| **4** | 9.305 | 0.1291091 | 8.513214 | 0.2524287 | 9.023889 | 0.1622133 | 8.759375 | 0.1328109 |
| **5** | 8.8075 | 0.1599544 | 8.666429 | 0.2624936 | 9.011666 | 0.1635877 | 8.736875 | 0.2056731 |
| **6** | 8.774375 | 0.176998 | 7.687143 | 0.1957388 | 8.616667 | 0.1038003 | 9.00375 | 0.146986 |
| **7** | 8.886875 | 0.1643109 | 6.437143 | 0.4687597 | 8.677222 | 0.1737379 | 7.975313 | 0.2935683 |
| **8** | 8.940938 | 0.1437896 | 5.762857 | 0.3631478 | 8.469444 | 0.1406439 | 7.430625 | 0.2030732 |
| **9** | 8.28875 | 0.1563247 | 6.610714 | 0.4139909 | 8.681111 | 0.1610076 | 7.89875 | 0.2322784 |
| **10** | 8.626875 | 0.1314082 | 6.669286 | 0.2708806 | 9.077778 | 0.1406813 | 8.300625 | 0.1570575 |
| **11** | 8.6875 | 0.2571276 | 6.236429 | 0.3524773 | 8.808333 | 0.1535128 | 7.828125 | 0.2565382 |
