# Supplementary material for: Regulatory role of capsaicin-sensitive peptidergic sensory nerves in the proteoglycan-induced autoimmune arthritis model of the mouse
Source: J Neuroinflammation. 2018 Dec 3;15:335. doi: 10.1186/s12974-018-1364-5 (PMC6276168; doi:10.1186/s12974-018-1364-5)
Supplement: Supplementary file 3 — Table S2. Mean and SEM values of the latency to fall from the horizontal grid of each experimental group. (DOCX 21 kb) [file 12974_2018_1364_MOESM3_ESM.docx]

**Table S2: Mean and SEM values of the latency to fall from the horizontal grid of each experimental group.**

| **days** | **non-desensitized control** | | **non-desensitized PGIA** | | **desensitized control** | | **desensitized PGIA** | |
| --- | --- | --- | --- | --- | --- | --- | --- | --- |
|  | **mean** | **SEM** | **mean** | **SEM** | **mean** | **SEM** | **mean** | **SEM** |
| **45** | 60.00 | 0.00 | 60.00 | 0.00 | 60.00 | 0.00 | 60.00 | 0.00 |
| **48** | 60.00 | 0.00 | 60.00 | 0.00 | 60.00 | 0.00 | 60.00 | 0.00 |
| **50** | 60.00 | 0.00 | 56.57 | 2.57 | 60.00 | 0.00 | 60.00 | 0.00 |
| **52** | 60.00 | 0.00 | 53.00 | 7.00 | 60.00 | 0.00 | 57.38 | 2.63 |
| **55** | 60.00 | 0.00 | 51.00 | 7.07 | 60.00 | 0.00 | 43.75 | 6.50 |
| **57** | 60.00 | 0.00 | 54.00 | 6.00 | 60.00 | 0.00 | 55.25 | 4.75 |
| **59** | 60.00 | 0.00 | 51.71 | 6.39 | 60.00 | 0.00 | 55.50 | 4.50 |
| **62** | 60.00 | 0.00 | 49.29 | 6.42 | 60.00 | 0.00 | 49.00 | 5.56 |
| **64** | 60.00 | 0.00 | 47.29 | 6.21 | 60.00 | 0.00 | 60.00 | 0.00 |
| **66** | 60.00 | 0.00 | 47.00 | 6.81 | 60.00 | 0.00 | 60.00 | 0.00 |
| **69** | 60.00 | 0.00 | 49.14 | 7.17 | 60.00 | 0.00 | 60.00 | 0.00 |
| **71** | 60.00 | 0.00 | 54.71 | 5.29 | 60.00 | 0.00 | 60.00 | 0.00 |
| **73** | 60.00 | 0.00 | 45.00 | 9.69 | 60.00 | 0.00 | 60.00 | 0.00 |
| **76** | 60.00 | 0.00 | 47.71 | 7.10 | 60.00 | 0.00 | 56.88 | 3.13 |
| **78** | 60.00 | 0.00 | 47.57 | 8.11 | 60.00 | 0.00 | 60.00 | 0.00 |
| **80** | 60.00 | 0.00 | 46.14 | 8.95 | 60.00 | 0.00 | 60.00 | 0.00 |
| **83** | 60.00 | 0.00 | 40.71 | 9.29 | 60.00 | 0.00 | 55.88 | 2.70 |
| **85** | 60.00 | 0.00 | 45.71 | 9.31 | 60.00 | 0.00 | 60.00 | 0.00 |
| **87** | 60.00 | 0.00 | 44.71 | 9.90 | 60.00 | 0.00 | 60.00 | 0.00 |
| **90** | 60.00 | 0.00 | 46.00 | 9.35 | 60.00 | 0.00 | 60.00 | 0.00 |
| **92** | 60.00 | 0.00 | 38.57 | 9.43 | 60.00 | 0.00 | 60.00 | 0.00 |
| **94** | 60.00 | 0.00 | 48.57 | 8.36 | 60.00 | 0.00 | 51.25 | 5.98 |
| **97** | 60.00 | 0.00 | 38.57 | 9.02 | 60.00 | 0.00 | 60.00 | 0.00 |
| **99** | 60.00 | 0.00 | 44.14 | 9.28 | 60.00 | 0.00 | 51.63 | 6.50 |
| **101** | 60.00 | 0.00 | 43.86 | 10.45 | 60.00 | 0.00 | 60.00 | 0.00 |
| **104** | 60.00 | 0.00 | 45.00 | 9.76 | 60.00 | 0.00 | 60.00 | 0.00 |
| **106** | 60.00 | 0.00 | 44.29 | 10.18 | 60.00 | 0.00 | 60.00 | 0.00 |
| **108** | 60.00 | 0.00 | 37.43 | 9.70 | 60.00 | 0.00 | 60.00 | 0.00 |
| **111** | 60.00 | 0.00 | 42.57 | 9.90 | 60.00 | 0.00 | 60.00 | 0.00 |
| **113** | 60.00 | 0.00 | 44.14 | 10.25 | 60.00 | 0.00 | 58.25 | 1.75 |
| **115** | 60.00 | 0.00 | 42.29 | 9.45 | 60.00 | 0.00 | 57.88 | 2.13 |
