# Supplementary material for: Regulatory role of capsaicin-sensitive peptidergic sensory nerves in the proteoglycan-induced autoimmune arthritis model of the mouse
Source: J Neuroinflammation. 2018 Dec 3;15:335. doi: 10.1186/s12974-018-1364-5 (PMC6276168; doi:10.1186/s12974-018-1364-5)
Supplement: Supplementary file 4 — Table S3. Results of the statistical analysis with repeated measures two-way ANOVA of mechanical hyperalgesia, paw edema, arthritis score, and optical imaging data of MPO activity and vascular leakage. (DOCX 14 kb) [file 12974_2018_1364_MOESM4_ESM.docx]

**Table S3: Results of the statistical analysis with repeated measures two-way ANOVA of mechanical hyperalgesia, paw edema, arthritis score, as well as optical imaging data of MPO activity and vascular leakage.**

|  | **non-desensitized control vs non-desensitized PGIA** | | **desensitized control vs desensitized PGIA** | | **non-desensitized PGIA vs desensitized PGIA** | |
| --- | --- | --- | --- | --- | --- | --- |
|  | **Group Interaction** | **Time** | **Group Interaction** | **Time** | **Group Interaction** | **Time** |
| **Mechanical hyperalgesia** | < 0.0001 | < 0.0001 | < 0.0001 | < 0.0001 | < 0.0001 | < 0.0001 |
| **Paw edema** | < 0.0001 | < 0.0001 | < 0.0001 | < 0.0001 | 0.006 | < 0.0001 |
| **Arthritis score** | < 0.0001 | < 0.0001 | < 0.0001 | < 0.0001 | 0.0007 | < 0.0001 |
| **MPO-activity** | < 0.0001 | < 0.0001 | 0.0107 | 0.022 | 0.0611 | < 0.0001 |
| **Vascular leakage** | < 0.0001 | < 0.0001 | < 0.0001 | < 0.0001 | 0.2736 | < 0.0001 |
